# Fluorescence relaxation kinetics of poly(methylphenylsilane) film and nanocomposites

**DOI:** 10.1186/s11671-016-1368-y

**Published:** 2016-04-12

**Authors:** N. Ostapenko, V. Gulbinas, R. Augulis, A. Boiko, M. Chursanova, A. Volkov, G. Telbiz

**Affiliations:** Institute of Physics of NASU, pr. Nauki 46, Kiev, 03028 Ukraine; Center for Physical Sciences and Technology, Savanoriu 231, Vilnius, Lithuania; National Technical University “Kyiv Polytechnic Institute”, Pr. Peremohy 37, Kyiv, 03056 Ukraine; Institute of Physical Chemistry NASU, Pr. Nauky 31, 03039 Kyiv, Ukraine

## Abstract

A comparative study of fluorescence relaxation kinetics of σ-conjugated poly(methylphenylsilane) (PMPS) polymer film and nanocomposites has been performed by ultrafast time-gated fluorescence measurements at various temperatures. Investigations have revealed a fine structure of excitonic σ-σ* band. We attribute this structure to emission from two spatially independent states with different ordering of the polymer chain segments, type *gauche* and *trans* conformations. In contrary to a more ordered polymer poly(di-*n*-hexylsilane), no clear thermochromic transition has been detected in PMPS film; however, the *trans* band intensity increases with temperature and with excitation wavelength, but it is absent when polymer is incorporated into nanopores of small diameter.

## Background

Polysilanes belong to the class of silicon-organic polymers consisting of σ-conjugated Si backbone and organic side groups. Their electronic properties are attributed to the σ-conjugation originating from the overlap of Si sp3 orbitals [1]. Polysilanes show remarkable fluorescence (FL) in the UV region [1] and high mobility of holes [2]; thus, these polymers are promising in construction of emitting or transporting layers for electroluminescence devices [3–5]. Evidently, structural organization of these polymers in solid state predetermines functioning of polymer-based devices; therefore, understanding of optical and electric features depending on the polymer structural arrangement is an important issue. Current trends in designing nanostructured materials for various applicative purposes can be achieved by means of different technological approaches. Embedding of polysilanes in nanoporous materials such as MCM-41 and SBA-15 is an effective way of producing and controlling nanostructured composites [6–9].

The shape of σ-σ* absorption band of polysilanes is determined by the *trans-gauche* isomerism of polymer chains and by the length distribution of conjugated segments [1]. Such isomerism has been clearly observed in poly(di-*n*-hexylsilane) (PDHS) polymers with clear thermochromic transition from *trans* to *gauche* conformation at about 315 K temperature [10]. Conjugated segments of different lengths have different transition energies; therefore, irradiation of the polymer into the blue edge of the absorption band preferentially excites the short higher-energy segments. Then, over their lifetime, excitons migrate to the longest segments, which have the smallest band gap and the lowest energy. Irradiation into the red edge of the absorption band addresses longer segments, increasing correlation between the absorbing and the emitting species. Thus, position of the FL band maximum in both cases is determined by the transition energies in the longest segments. FL quantum yields of polysilanes also depend on the excitation wavelength and reach their maximum values for the long-wavelength excitation [1]. Temperature dependences of the FL spectra measured with high time resolution may provide information about intermediate stages. Relaxation processes are expected to be more complex in the case of coexistence of different conformeric forms.

This article presents ultrafast time-resolved fluorescence study of excited state dynamics of σ-conjugated poly(methylphenylsilane) (PMPS) films and PMPS confined within MCM-41 and SBA-15 silica nanopores of 2.8 and 9 nm diameter, respectively. Investigation reveals coexistence of spatially separated *trans* and *gauche* conformational forms of polymer chain, and we conclude that the lower energy *trans* states are partly populated by thermally stimulated exciton diffusion.

## Methods

PMPS polymer was synthesized as described in [1]. PMPS films (Mw = 11160) were prepared by drop-casting from the solution in toluene (1 wt.%) on metal substrates. Silicas MCM-41 and SBA-15 with the pore diameter of 2.8 and 9 nm, respectively, were synthesized by the techniques given in [11, 12]. The synthesized samples of MCM-41 were filtered and washed and then carefully calcined in air at heating rate of 1 K/min up to 813 K and held at this temperature for 5 h. In the case of SBA-15, the samples were calcined in an oven at 373 K with the heating rate of 1 K/min for 3 h and, subsequently, in dry air at 823 K for 4 h to remove the surfactant. The removal of the template was controlled by FTIR spectroscopy.

Mesoporous material was mixed with the polymer solution; afterwards, the mixture was placed into a dark vessel where it was slowly stirred for 2 days at 293 K and kept until the solvent evaporated. The obtained nanocomposite was double washed in fresh toluene to remove the polymer from the outer surface. Then, the obtained samples were dried for 12 h at room temperature for the residual moisture to be removed and then they were kept in a desiccator.

Steady-state FL spectra of PMPS films were measured with spectrometer based on streak camera described below, while FL spectrum of solution in toluene at 15 K temperature was measured with DFS-13 spectrometer under excitation at 313 nm. Ultrafast fluorescence dynamics of PMPS films and nanocomposites was measured by means of spectrometer based on a streak camera (Hamamatsu Photonics Ltd.) and femtosecond laser (Light Conversion Ltd.). The Yb:KGW laser produced 80-fs 1030-nm light pulses at 76 MHz repetition rate. Third (343 nm) and fourth (254 nm) harmonics of the laser radiation generated by HIRO harmonics generator (Light Conversion Ltd.) were used for the sample excitation. The excitation beam was focused to ∼100 μm spot on the sample, resulting in about 1 mW/mm^2^ average excitation power. The maximum time resolution of the whole system was about 3 ps, and spectral resolution was about 3 nm. All the measurements (except of solution in toluene) were performed in closed cycle cryostat (Janis Research Company Ltd.), which enabled temperature variation within 15–320 K. The samples were kept in vacuum during the measurements.

## Results and Discussion

**Fig. 1:**
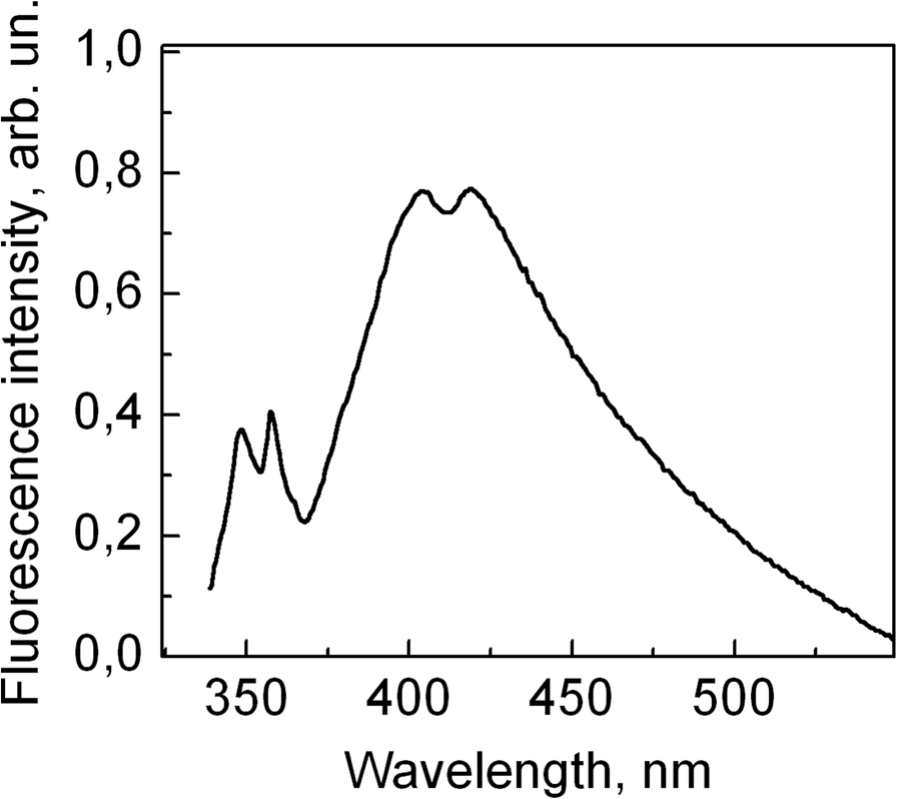
Fluorescence spectrum of PMPS solution in toluene with 10^−2^ mol/l concentration at 15 K temperature

According to the literature data, absorption spectrum of PMPS film at room temperature has two bands at 338 and 270 nm, which correspond to σ-σ* excitonic transition inside the backbone of polymer chain and π-π* transition within side phenyl groups, respectively [13]. Figure 1 presents fluorescence spectrum of matrix-isolated PMPS in toluene at 15 K. The spectrum consists of a narrow doublet band in UV region with maxima at 348 and 356 nm and a broad band in the visible region. The doublet short-wavelength band should be attributed to the σ*-σ transition of the polymer backbone chain. Its splitting suggests coexistence of two conformational states of isolated polymer chains. It is known that PMPS present in less ordered form in liquid solution tends to stretch upon cooling adopting a more ordered morphology [14]. Apparently, a fraction of the polymer chains at 15 K does acquire the partially ordered conformation causing appearance of the fluorescence band at 356 nm.

**Fig. 2:**
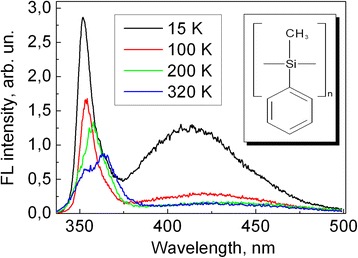
Steady-state fluorescence spectra of PMPS film at different temperatures measured under excitation at 254 nm. The molecular structure of PMPS is illustrated in the *inset*

Steady-state FL spectra of PMPS film at various temperatures measured under excitation at 254 nm are presented in Fig. 2. As well as for the matrix-isolated polymer, it shows UV and visible fluorescence band; however, we do not observe the band splitting at low temperature. The origin of the visible luminescence band has been widely discussed [13, 15–22]. It has been suggested that it actually consists of two bands with maxima at about 410 and 450 nm. Two possible explanations of the 410 nm band have been suggested: σ-π* intramolecular charge-transfer (CT) emission [15, 16] and a backbone defect emission [13, 17–21]. The weak band at 450 nm is associated with defects in the polymer chain such as cross-link points or so-called weak bond formed by the photolysis of the polymer [19, 20, 22].

As temperature increases up to 200 K, a new UV band gradually appears at 357 nm, but a weak short-wavelength shoulder remains in the position of the band observed at 15 K. At room temperature, the UV band acquires clear doublet structure.

**Fig. 3:**
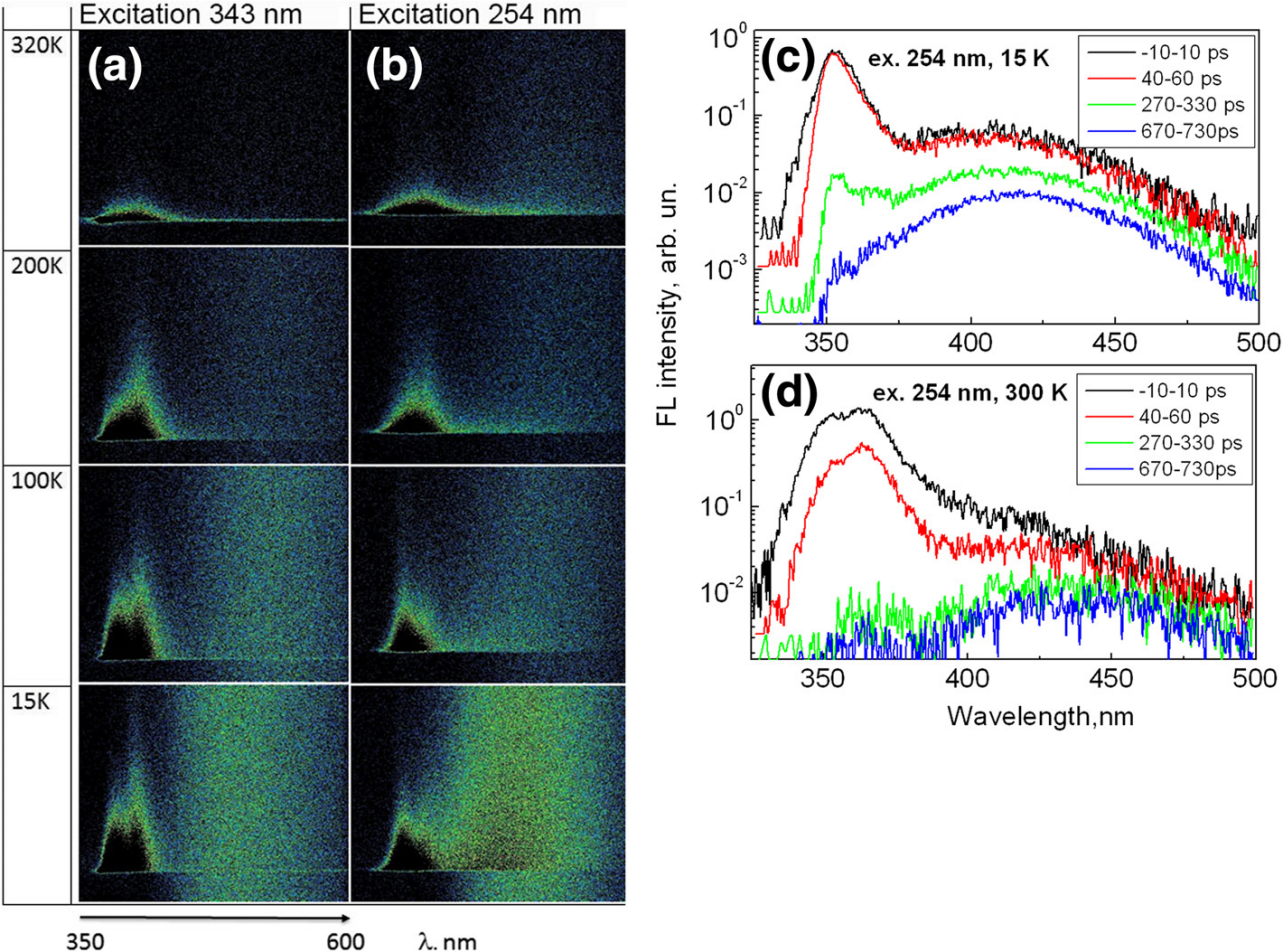
Streak camera data for the time-evolution of PMPS film fluorescence at different temperatures obtained under excitation at 343 nm (**a**) and 254 nm (**b**). Graphs (**c**) and (**d**) show fluorescence spectra averaged over several time intervals at 15 and 320 K under excitation at 254 nm

Figure 3a, b shows streak camera data for the time-resolved fluorescence spectra of PMPS film at different temperatures obtained under excitation at 343 and 254 nm. In contrast to the time-integrated spectra, the time-resolved streak camera data clearly show the existence of two excitonic bands even at all temperatures. Within experimental accuracy, the band positions are temperature independent; however, their relative intensities depend on temperature and excitation wavelength. Figure 3c, d shows FL spectra integrated over several time intervals at 15 K and at room temperature under excitation at 254 nm. For more clear representation of very different intensity spectra obtained at different times, they are presented in log scale. The initial FL spectrum measured at 15 K, as well as the steady-state spectrum, shows a narrow intense UV band; however, its maximum appears at a slightly shorter, 352-nm wavelength. The shift is within spectral resolution of the instrument; on the other hand, the shift between the initial and steady-state FL spectra is commonly observed in molecular solids because of gradual exciton localization at low-energy sites. Intensity of the UV band drops down at 300 ps and reveals the presence of a shoulder at about 365 nm. With the further increase of delay time, intensity of the UV band decreases, and basically, only the broad visible band shifted to about 420 nm is observed. At higher temperatures, the doublet structure of the exciton band becomes more pronounced. We clearly observe two excitonic band components at 320 K. The short-wavelength component is approximately at the same spectral position as at 15 K, and the long-wavelength component has a peak at about 364 nm. As well as the short-wavelength component, it is also slightly shifted to the short-wavelength side in comparison with the long-wavelength band in the steady-state spectrum.

**Fig. 4:**
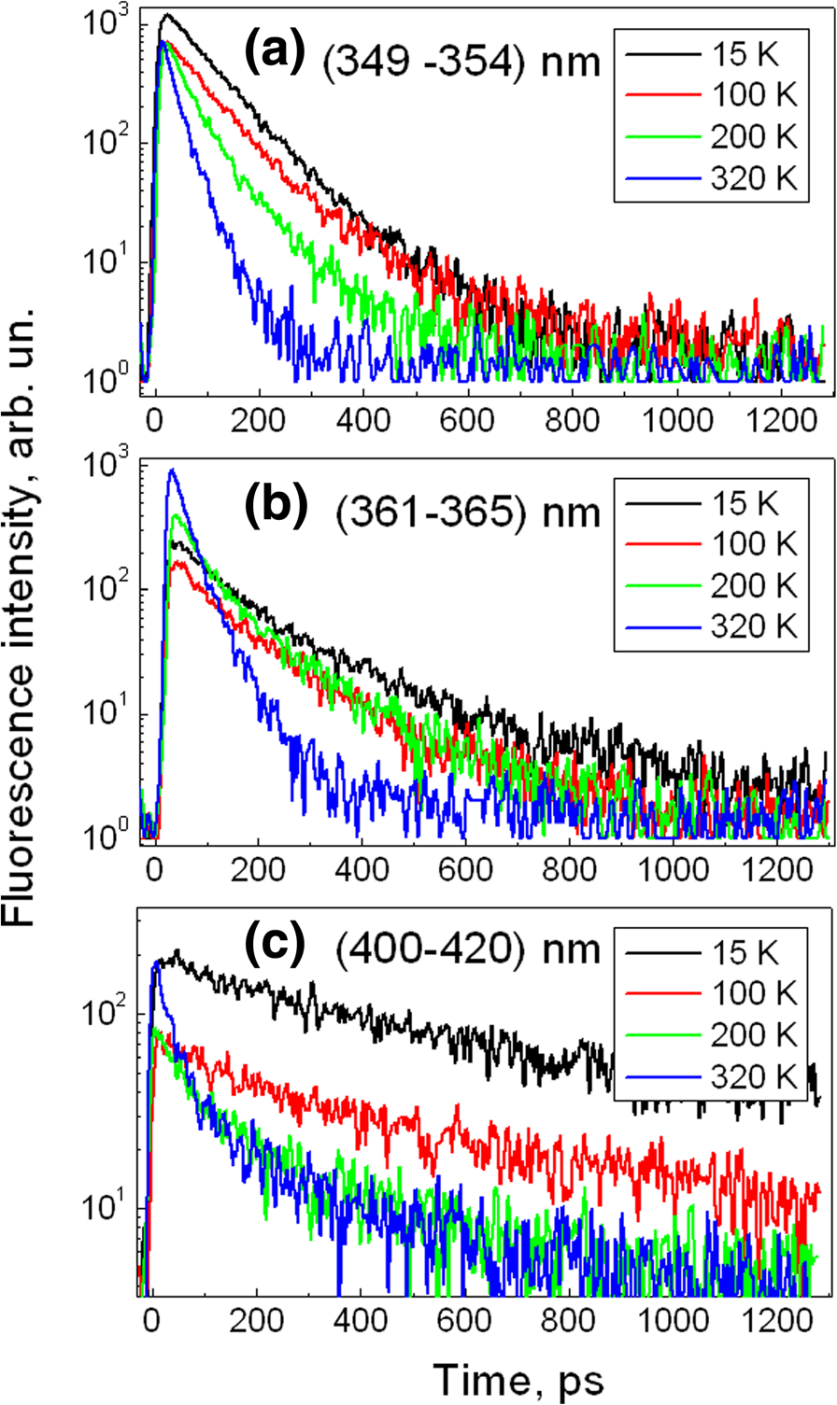
Temperature dependences of the fluorescence decay kinetics measured in spectral regions of the short-wavelength (**a**), long wavelength (**b**) excitonic bands, and visible fluorescence band (**c**) under excitation at 254 nm

**Fig. 5:**
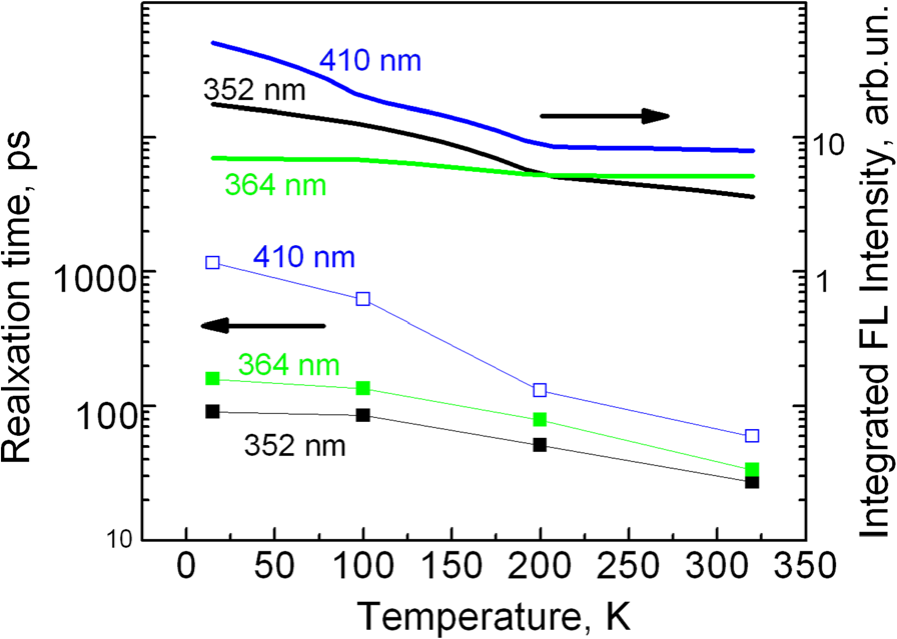
Relaxation times and amplitudes for doublet components of the exciton band under excitation at 254 nm

Figure 4 shows decay kinetics of different fluorescence bands of PMPS film measured at different temperatures. For better accuracy, the kinetics is averaged over spectral regions specified in Fig. 4. The decay kinetics may be well approximated by biexpoenentail decay functions; however, amplitude of the slow component is much lower, and therefore, only the fast decay time was taken into account. Figure 5 summarizes the data on temperature dependences of relaxation times of the doublet components and of the visible luminescence band. The short-wavelength excitonic doublet component decays slightly faster than the long-wavelength component and their lifetimes decrease with temperature. Decay of the visible luminescence is several times slower. Decay of the long-wavelength excitonic component (with the time constant of 88 ps) measured at 15 K agrees well with its relaxation rate at 2 K reported in ref. [23]. As Fig. 5 shows, all relaxation times decrease with temperature, but relaxation time of the visible band at 410 nm decreases particularly strongly, more than ten times (take note of vertical logarithmic scale). Strong temperature dependence of the 410-nm band suggests that there are some additional fluorescence quenchers which quench the 410-nm band at higher temperatures when exciton migration becomes more efficient. Perhaps, it is a defect state at 450 nm. Figure 5 also shows temperature dependences of the time-integrated intensities of the three FL bands. Intensity of the 410-nm band decreases with temperature more significantly than the intensity of the 352-nm band, while intensity of the 364-nm band is almost temperature independent. Apparently, excitation energy is transferred from the species luminescing at 352 nm to the species luminescing at 364 nm, and this transfer is more efficient at higher temperatures.

We suppose that the two excitonic bands are associated with the existence of spatially separated FL centers, which correspond to different ordering of the polymer chain segments, i.e., to different chain conformations, of *gauche* and *trans* types. Such polymorphism has been clearly observed for PDHS polymers causing the appearance of two fluorescence bands: the short-wavelength band attributed to the disordered *gauche* conformation and the long-wavelength band attributed to a more ordered *trans* conformation [24]. Existence of *gauche* and *trans* conformational forms of PMPS film was also suggested in ref. [25]; however, properties of the two forms were not investigated in detail. Existence of similar two states in steady-state FL spectrum of thick PMPS film was also proposed in ref. [26]. Our results show interplay between the fluorescence of the *gauche* and *trans* forms. Increase of intensity of the long-wavelength component with respect to the short-wavelength component as temperature increases from 15 to 320 K suggests that the *trans* species are at least partly populated via thermally activated energy transfer from the *gauche* species. *Gauche* species are apparently dominantly excited with 254 nm light and excitation energy transfer to *trans* species at low temperatures, up to about 200 K, is inefficient. *Trans* species, according to the ref. [27], have absorption band at about 341 nm; thus, they are efficiently excited with 343 nm light and, consequently, *trans* fluorescence is more intense at all temperatures (Fig. 3a).

**Fig. 6:**
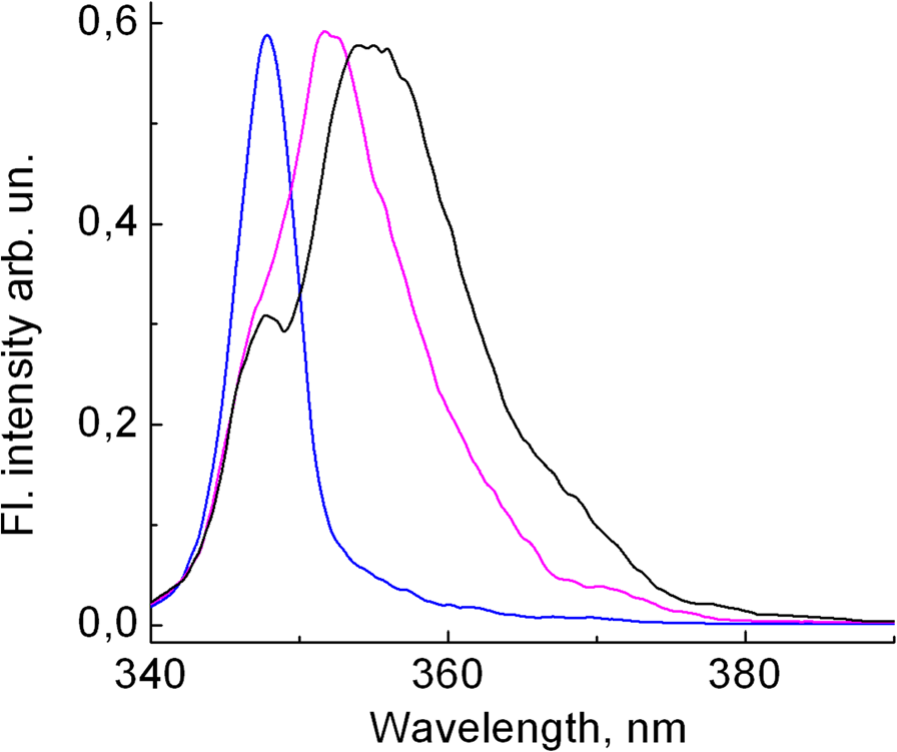
FL spectra integrated during 400 ps of PMPS/MCM-41 nanocomposites at *T* = 15 (*blue*) and of PMPS/SBA-15 nanocomposites at *T* = 15 (*magenta*) and at *T* = 200 K (*black*) measured under excitation at 343 nm

Incorporation of polymer into silica nanopores gives another option of partial isolation and ordering of polymer chains. Polymer incorporated into silica nanopores of different diameter allows us to detect transition from isolated macromolecules to polymer film. Such transformation via aggregate formation has been observed for PDHS/SBA-15 nanocomposites [24]. Small diameter (2.8 nm) pores of MCM-41 matrix may contain only two macromolecules. In that case, the intermolecular interaction between macromolecules is considerably weakened with respect to that in the polymer film because macromolecules may have only one neighbor. So, the σ*-σ transition of the isolated polymer chain is expected to be shifted towards the short-wavelength region with respect to that in the film. Indeed, as Fig. 6 shows (blue curve), FL spectrum of PMPS/MCM-41 nanocomposite at 15 K has very narrow exciton band at 348 nm shifted to a shorter-wavelength region by about 4 nm with respect to that in the film, and its position coincides with the *gauche* band position in the toluene matrix (see Fig. 1). The exciton band in FL spectrum of PMPS/SBA-15 nanocomposite at 15 K is significantly broadened with respect to the corresponding band of PMPS/MCM-41 nanocomposite and consists of two bands. In addition to the *gauche* band, which coincides with the corresponding band of PMPS/MCM-41 composite, a new long-wavelength *trans* band appears at 352 nm (Fig. 6, magenta curve). As temperature increases up to 200 K, the *trans* band shifts to longer wavelengths (Fig. 6, black curve) and coincides with the corresponding band in spectrum of PMPS film at 200 K, as well as with the *trans* band in the toluene matrix. The position of the *gauche* band remains unchanged. Pore diameters of SBA-15 matrix are much larger, of 9 nm, and therefore, it may host larger number of polymer chains. So, a larger shift suggests that aggregated states of *trans* conformation polymer chains may be formed in SBA-15 matrix as well as in neat films. Formation of aggregate states with red-shifted fluorescence was clearly observed for PDHS/SBA-15 nanocomposite [24].

Although PMPS, as well as PDHS polymer, reveals two conformeric states, their properties are rather different. PDHS embedded in nanoporous silica shows clear thermochromic transition from *gauche* to *trans* form in a (220-290) K range [24], while we do not see any evidence of thermochromism of PMPS. The difference is apparently related to the polymer structure. In contrast to PDHS, which is semicrystalline polymer, PMPS is an amorphous material; therefore, it has no distinct phase transitions, and the interplay between the two conformeric forms sufficiently clearly appears only in time-resolved fluorescence spectra. Consequently, our data reveal coexistence of *gauche* and *trans* conformeric forms in PMPS films independently of temperature. Although the *gauche* fluorescence in films decays up to two times faster than the *trans* fluorescence, energy transfer between the two conformers is not efficient suggesting that *trans* conformers are in minority and/or *trans* and *gauche* forms are spatially separated. *Gauche* conformers completely dominate when PMPS is incorporated in porous materials with small pore diameter, while in larger pores, the *trans* conformers and probably their aggregates are formed as well.

## Conclusions

Conformational properties and excited state relaxation of σ-conjugated poly(methylphenylsilane) (PMPS) polymer film and nanocomposites have been investigated at different temperatures by ultrafast time-gated fluorescence measurements. PMPS shows exciton band in the UV region and a wide visible fluorescence band in the 400- to 500-nm regions. PMPS exciton fluorescence has doublet structure in nanocomposites and in low-temperature toluene matrix. Time-resolved investigations at different temperatures clear revealed the doublet structure and in PMPS film. We attribute this structure to the coexistence of *gauche* and *trans* conformations of polymer chain. Intensity of the long-wavelength component of the exciton band attributed to the *trans* conformation increases with the temperature and with excitation wavelength indicating partial population of this state via thermally activated energy transfer. The long-wavelength component attributed to the *trans* conformation is absent in the composite with small pore diameter hosting only one or two polymer chains where exciton diffusion is hindered. In the composite with larger pore diameters, the *trans* conformers and probably their aggregates are formed as well. In contrast with the semicrystalline PDHS, no thermochromic *trans-gauche* transitions are observed in PMPS because of its amorphous structure.
